# Implementation of a School-Based Social and Emotional Learning Intervention: Understanding Diffusion Processes Within Complex Systems

**DOI:** 10.1007/s11121-015-0552-0

**Published:** 2015-03-01

**Authors:** Rhiannon Evans, Simon Murphy, Jonathan Scourfield

**Affiliations:** DECIPHer, School of Social Sciences, Cardiff University, Cardiff, UK

**Keywords:** Social and emotional learning, Intervention, Implementation, Diffusion of innovations

## Abstract

Sporadic and inconsistent implementation remains a significant challenge for social and emotional learning (SEL) interventions. This may be partly explained by the dearth of flexible, causative models that capture the multifarious determinants of implementation practices within complex systems. This paper draws upon Rogers (2003) Diffusion of Innovations Theory to explain the adoption, implementation and discontinuance of a SEL intervention. A pragmatic, formative process evaluation was conducted in alignment with phase 1 of the UK Medical Research Council’s framework for Developing and Evaluating Complex Interventions. Employing case-study methodology, qualitative data were generated with four socio-economically and academically contrasting secondary schools in Wales implementing the Student Assistance Programme. Semi-structured interviews were conducted with 15 programme stakeholders. Data suggested that variation in implementation activity could be largely attributed to four key intervention reinvention points, which contributed to the transformation of the programme as it interacted with contextual features and individual needs. These reinvention points comprise the following: intervention training, which captures the process through which adopters acquire knowledge about a programme and delivery expertise; intervention assessment, which reflects adopters’ evaluation of an intervention in relation to contextual needs; intervention clarification, which comprises the cascading of knowledge through an organisation in order to secure support in delivery; and intervention responsibility, which refers to the process of assigning accountability for sustainable delivery. Taken together, these points identify opportunities to predict and intervene with potential implementation problems. Further research would benefit from exploring additional reinvention activity.

## Background

Social and emotional learning (SEL) has been linked to a range of mental health, behavioural and educational outcomes (Qualter et al. 2012; Zins et al. 2007). A plethora of school-based interventions have been developed in the attempt to develop children and young people’s social and emotional competencies, with approaches including: the targeting of individuals exhibiting high risk behaviours (Kendal et al. 2011); the systematic teaching of skills within the classroom (Greenberg et al. 1995); and complex, whole-school approaches that seek to engage in contextual restructuring (Bond et al. 2004). Systematic reviews and meta-analyses have demonstrated the effectiveness of intervention in this area (Weare and Nind 2011), with Durlak et al.’s (2011) comprehensive study of 213 programmes finding a grand study-level mean of 0.30 (95%CI = 0.26–0.33) for outcomes including SEL skills, attitudes, positive social behaviour, conduct problems, emotional distress and academic performance.

Despite this evidence, intervention outcomes have been compromised by sporadic and inconsistent implementation (Durlak and DuPre 2008; Greenberg 2010; Spoth et al. 2013; Wandersman et al. 2008). This is largely a consequence of barriers to adequate delivery or programme adaptation in the effort to ensure cultural congruence (Lendrum and Humphrey 2012). Sustainable implementation has provided a further challenge, with Elias et al. (2000) warning of schools’ tendency to treat interventions as inoculations rather than long-term prevention plans. Implementation continues to receive limited monitoring within evaluation however (Durlak et al. 2011; Lendrum and Humphrey 2012), and where implementation checks are integrated, they routinely fail to adopt a multidimensional approach (Domitrovich and Greenberg 2000). Yet, the necessity of attending to variability in implementation practices is evident, as this arguably constitutes the most significant moderator of outcomes (Dane and Schneider 1998; Durlak et al. 2007). In Banerjee’s (2010) evaluation of the Social and Emotional Aspects of Learning (SEAL) programme in England, 49.8 % of school-level variance in academic attainment could be accounted for by differences in implementation and the social and emotional ethos of schools.

Central to the failure to sufficiently address variable implementation within both praxis and evaluation has been the dearth of flexible, causative models that possess the explanatory power to help understand complex implementation processes. There has been limited differentiation between different determinants, needs and problems during different phases of implementation activity (Lendrum and Humphrey 2012). There also remains a propensity to treat implementation as a discreet phenomenon that is determined in situ, rather than part of a chronology of activity that precedes and proceeds delivery (Spoth et al. 2013). Equally, interventions are often artificially disentangled from the setting in which they are delivered, leading Bauman et al. (1991, p. 620) to astutely observe a much ‘ignored sociological proposition that organisational innovation in general, and new programs in particular, do not exist as a separate entity independent of context’.

An increasing number of empirical studies, and synthesis of research evidence, have sought to engage with the challenges of implementation. Prior to adoption, there is a critical need for programme credibility, accessible information demonstrating intervention utility, and well-connected and respected champions (Durlak and DuPre 2008; Elliott and Mihalic 2004; Wandersman et al. 2008). Addressing preparedness to change, through consultation with organisations, is required to ensure sufficient structures for sustainable practices (Greenberg 2010). Training quality has also been emphasised, with a recognised need to provide relevant information, demonstrations, and opportunities for behavioural rehearsal (Fixsen et al. 2005). Organisational capacity and commitment remain a vital factor, particularly in terms of support systems, effective leadership, and dedication of resources, climate and culture (Greenberg 2010; Greenhalgh et al. 2004; Humphrey et al. 2010; Kam et al. 2003). However, although these studies have made a substantial contribution to the understanding of intervention delivery, they have tended to be empirically driven and focus on discrete aspects of implementation. As a result, the complex interaction of individual and contextual determinants of implementation is not always fully considered or understood. This suggests the need for a more comprehensive theoretical frame to structure the empirical exploration of intervention practices within real-world settings.

Rogers’s (2003) diffusion of innovations theory has much to offer here, providing a comprehensive conceptual framework for understanding how implementation is determined by the complicated and often protracted interaction of an intervention with contextual features and individual needs. This theory has increasingly gained traction within SEL and prevention interventions more broadly (Durlak and DuPre 2008). It has been used in the Interactive Systems Framework for Dissemination and Implementation (ISF) of Wandersman et al. (2008) to enhance the application of research evidence within real-world settings. Most recently, it has been employed as part of the Translation Science to Population Impact (TSci Impact) Framework of Spoth et al. (2013), which is a heuristic tool offering key considerations for improving the translation of evidence-based interventions.

Defined as ‘the process in which an innovation is communicated through certain channels over time among the members of a society’ (Rogers 2003, p. 5), diffusion comprises five constituent phases. The first phase addresses adoptive organisations’ *knowledge* of an intervention with regard to compatibility, complexity, relative advantage, trialability and observability. Second is *persuasion* to adopt a programme. Persuasion is generally initiated by a change agent who serves as an intermediary between the change agency (e.g. intervention developer) and the adopter, seeking to influence an innovation-decision in a direction favouring change. Change agents may be external to an organisation but can be more effective when drawn from the adoptive context due to their homophily with other members. Third is *adoption* and the decision to use an intervention as the best course of action available. The duration of this phase is largely determined by the decision approach employed. In hierarchically structured organisations, such as schools, an authority innovation-decision is often made, whereby adoption is determined by relatively few individuals who possess power. In this instance, disempowered organisational members may resist the challenges to structures that once provided a semblance of stability and continuity. Rogers (2003) suggests that a clarification period may be required so that an intervention becomes gradually clearer to system members and is not interpreted as a top down imposition. Fourth is the *implementation* phase, which sees the intervention transition from a mental exercise of hypothesising to invoking real change. This necessitates skill development and ongoing assistance from the change agency (Wandersman et al. 2008; Zins et al. 2004). Fifth is intervention *confirmation*, whereby the adopter decides to continue with an intervention, continue with reinvention or completely discontinue.

A pragmatic, formative process evaluation was conducted of a non-evidence-based SEL intervention, the Student Assistance Programme (SAP), with the aim to understand and refine the intervention so that it may be later subjected to rigorous scientific evaluation for effectiveness. The study sought to elicit the intervention’s theoretical underpinnings, understand how the intervention was diffused, estimate existing levels of implementation and ascertain participants’ experiences. Drawing on Rogers’s (2003) diffusion of innovations as an organising theoretical framework, this paper explores the diffusion and implementation of the SAP. It specifically focuses on the development of the concept of ‘reinvention points’. The concept of reinvention is routinely employed within the diffusion literature to define the degree to which an intervention is changed or modified during diffusion processes (Rogers 2003). Reinvention points offer further development of this concept through the identification of *significant moments* where interventions are adapted (often unconsciously) as they interact with contextual features and individual needs. They essentially demarcate key levers to variations in implementation. The four key reinvention points to emerge during the diffusion of the SAP, and which are presented in this paper, include the following: intervention training, intervention assessment, intervention clarification and intervention responsibility. They mark transitory phases between Rogers’s (2003) diffusion of innovation stages, as these are when intervention practices are most unstable, and where programmes may be most susceptible to change.

### The Student Assistance Programme

The SAP is a school-based intervention aiming to improve children and young people’s social and emotional competencies in order to mitigate social, emotional and behaviour problems (Watkins 2008). Developed in the USA, it has been delivered in Wales since 2003 and has been recommended by the Welsh school inspectorate as best practice in managing challenging behaviour. There are no formal records of uptake in Wales, although the intervention author reports that approximately 100 of 1478 primary schools and 40 of 223 secondary schools have implementation experience.

The SAP is a complex, whole-school intervention comprising 12 inter-related components: SAP leadership and administration, which involves appointment of a change agent as a coordinator to lead on delivery; integration of the SAP into local authority and community policies and procedures; an advisory committee of school/community representatives to input expertise into SAP activity; education of school staff about SAP and well-being more broadly; improving staff wellness; education and support of parents and community; networking with the community to provide support and developmental opportunities to students; infusion of SEL activities into the curriculum; identification and referral procedures for student support groups; a student support group addressing the social and emotional competencies of targeted individuals exhibiting social, emotional or educational problems; and evaluation of the student support group by staff and students.

Diffusion processes commence with the intervention author and a voluntary national coordinator, who deliver all intervention training in Wales, inviting local organisations to attend a 3-day training course. Training is usually funded through the local education authority. Two to three members of school staff attend each training course, with more staff attending subsequent training. The four schools in the study had between three and ten staff trained, with no fixed plan to offer training to more. On schools receiving the invitation to attend, senior managers elect appropriate staff members (e.g. pastoral support staff) who express an interest. During the course, 1 day is spent communicating the academic research underpinning SEL, 1 day involves learning how to integrate the intervention into organisational settings and 1 day is spent simulating delivery of the support group. The intervention handbook structures these activities and contains materials on how to deliver all 12 components (Watkins 2008). Throughout this period, a number of ‘change agents’ emerge, who appoint themselves as champion of the intervention within their respective organisations, aiming to secure adoption and instigate implementation. All staffs are expected to engage in intervention delivery as implementation unfolds, although it is only mandatory that those directly involved in delivery of the student support group must attend training.

Limited evaluation of the SAP has been conducted, with most studies offering description of programme aims, implementation procedures and participant perspectives (Carnwell and Baker 2007; Porter 2009; Taylor and Baker 2012). Where pre-post-evaluation has been conducted, there have been reported improvements in self-awareness, self-regulation, social skills, motivation, empathy and overall emotional literacy (Porter 2009). In their qualitative evaluation, Carnwell and Baker (2007) consider how contextual features impact upon delivery, with pertinent factors encompassing the insufficient staffing of SEL interventions, limited funding that forces schools to prioritise other competing demands and a constrained curriculum that is geared towards academic skills.

## Method

This paper presents data from a pragmatic, formative process evaluation (Evans et al. 2014a, b) conducted in adherence to phase 1 of the UK Medical Research Council’s (MRC) guidance for the development and evaluation of complex interventions (Craig et al. 2008; MRC 2000). The MRC is a publicly funded government agency responsible for coordinating research into medical and related sciences in the UK. Case-study methodology was employed (Yin 1994). Data were generated from four mixed-sex secondary schools (aged 11–18) in Wales. Schools were drawn from two local education authorities. Three were from a post-industrial town: Ysgol-y-Dyffryn, Ysgol-y-Glyn and Ysgol-y-Cwm. The fourth was from a small rural town: Ysgol-y-Foryd. Schools were purposively selected if they implemented the SAP and were diverse in terms of socio-economic context (free school meal entitlement ranging from 11.3 to 36 %) and academic achievement (GCSE A*-C grades or equivalent qualification in core subjects ranging from 16.9 to 59.8 %). Data generated with purposively sampled programme stakeholders from the four schools are presented in this paper. Fifteen individuals were interviewed, comprising the following: the intervention author (*N* = 1), national coordinator (*N* = 1), senior school managers (*N* = 4) and change agents (*N* = 9). Change agents’ professional roles included the following: school nurse, teacher, truancy officer, learning support assistant, child psychologist, counsellor and youth worker.

Semi-structured interviews were conducted (Britten 2006). All interviews were undertaken by the study’s primary researcher. Interviews lasted from 40 min to 2 h. Discussions were structured by interview schedules, which provided a number of pre-determined but non-standardised questions. These reflected the aims of the process evaluation (Linnan and Steckler 2002), with questions around implementation being broadly structured by Rogers (2003) diffusion phases. Schedules were adapted according to the role of the professional. For example, senior managers were asked about their decision to invest organisational resources into the invention, while change agents were asked how and why they approached senior managers to secure investment. Interviews were digitally recorded and transcribed verbatim. All interviews were conducted at the participants’ workplace, and field notes were generated during these visits, with the consent of schools and individuals to capture additional reflections, conversations and observations.

Analysis drew upon a thematic approach (Strauss and Corbin 1990). Coding was conducted by the primary researcher and verified by two other members of the research team. Data were first coded to identify the presence of pre-specified themes. These themes mapped onto the questions being asked as part of the process evaluation, which included understanding how Rogers’s (2003) diffusion phases played out. A second broader reading of the data was taken in order to elicit novel themes (e.g. the role of personal transformation and belief). Codes predominantly deconstructed the data into short phrases, with parent codes representing an excerpt’s relevance to a general theme (e.g. implementation) and child codes representing its relevance to a particular strand within this theme (e.g. attitude of staff). Following codification, codes pertaining to similar themes were grouped together to generate sets of categories that related to each of the research questions. The reinvention points comprised the four key categories that explained the diffusion of the SAP. Data collection and analysis were conducted iteratively, with emergent themes being explored in later interview schedules. NVivo software supported the process of data analysis. Ethical approval was provided by Cardiff University’s School of Social Sciences Research Ethics Committee.

## Results

**Fig. 1:**
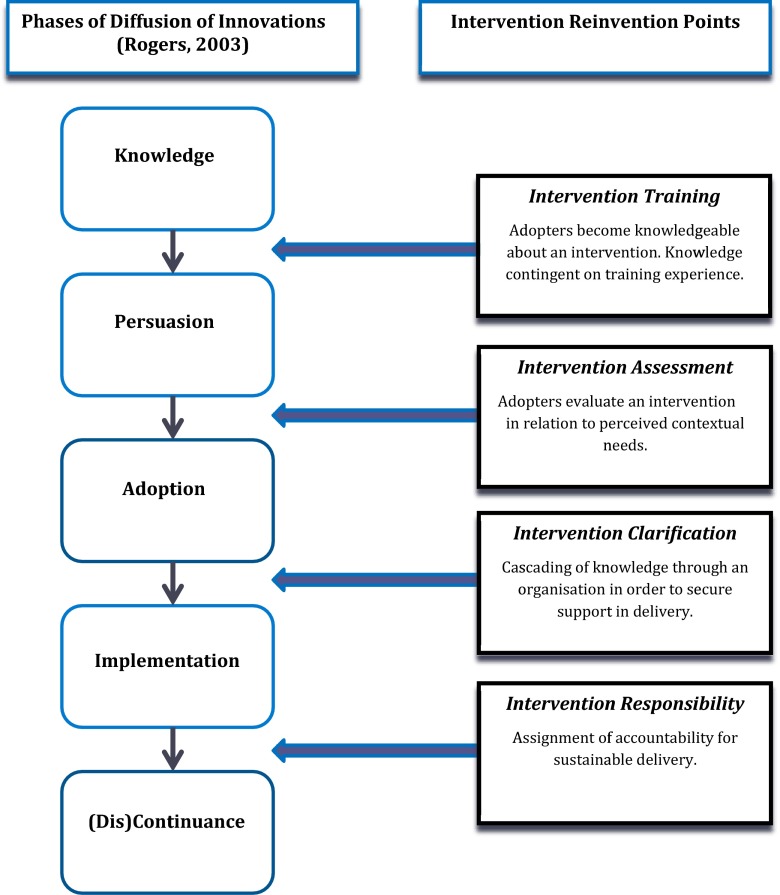
Reinvention points within the diffusion of innovations

The data indicated a low level of delivery of intervention components. However, limited implementation activity was not the consequence of an active decision on the part of schools. Indeed, discussions throughout the study revealed that adopting individuals and institutions perceived themselves to be fully implementing the Student Assistance Programme, with schools routinely classifying themselves as a ‘SAP school’. Rather, the intervention was subtly and often unconsciously reinvented as it transitioned through the diffusion phases, manifesting as (1) a complex intervention; (2) the student support group, organisational referral mechanisms and staff and student evaluation being the most immediate components to be taken up, amidst an intention to deliver the remaining components at a later date; (3) the student support group, organisational referral mechanisms and staff and student evaluation being the only components adopted; (4) the student support group constituting a peripheral intervention with no organisational referral mechanisms or support from staff members; and (5) the intervention being disbanded. The study identifies four key *reinvention points*: intervention training, intervention assessment, intervention clarification and intervention responsibility. Introduction of the concept of reinvention points is useful as they encapsulate the complex reality of delivering interventions while identifying key moments where implementation problems may arise. Equally, they go some way in resolving the discrepancy between organisations believing that they are providing an intervention and the low level of implementation actually being delivered; reinventions may be so incremental and even imperceptible that they may not be overtly known to delivery agents. The reinvention points and their relation to Rogers’s phases of the diffusion of innovations are presented in Fig. 1.

### Intervention Training

The first reinvention point constitutes schools’ transition from being unaware of the intervention to being knowledgeable about its form and content. Within the SAP, this knowledge was acquired during change agents’ attendance at the 3-day training course. For attendees, the motivation to champion the intervention within their respective organisations, securing adoption and instigating implementation, is largely attributable to the experience of participating in the student support group during the training, wherein individuals simulate delivery and participation. A number of change agents described this experience as both transformative and cathartic, allowing them to resolve issues pertaining to their own social and emotional well-being:

*But after the initial three days training, on the Friday, apart from being exhausted, and all cried out as they* say…*And it was like ‘Wow, this is pretty cool’. You just got someone to just talk to. I don’t know really. It really, really had an impact on me…It’s just a good way to get stuff off your chest. And I think afterwards then, you feel a little bit, I don’t know, like a weight has been lifted off your shoulders almost.*

(Change Agent, Ysgol-y-Glyn)

*I think it definitely gave me a different view on life…SAP made me realise that it was totally healthy to talk to someone…Yeah. I did think it was transformational and it did motivate me a lot.*

(Change Agent, Ysgol-y-Cwm)

In response to this experience, change agents expressed a desire to see the positive power of the student support group extended to students and indicated a determination to implement this component, plus the organisational referral mechanisms and evaluation:

*I could see how that, something like this could benefit some children with problems…Even those who haven’t got problems. I didn’t know I had problems before I went. It would be great if you could offer this to every single child in Year 7 because it, they would gain something from it.*

(Change Agent, Ysgol-y-Dyffryn)

While the predominance of the student support group during the training course served as a vital motivator, differential emphasis on intervention components ensured that this element garnered the most attention and became the focus of interest, with an indication that change agents would introduce other elements at a later stage. This process was heavily supported by a lack of technical knowledge to ensure the adoption and implementation of the full SAP. A number of change agents highlighted how the training, despite dedicating a day to the 12 components, had offered insufficient expertise to deliver such a complex intervention, and when they wanted to expand delivery in the future, they would need to return to the author for additional guidance:

*I don’t think there was enough on the training about the twelve step wheel. I find it’s very, what we’ve been told is very vague. And there’s a lack of understanding about the twelve steps to be honest with you. Um, obviously we want to push SAP as far as it will physically go. …We would need to sit down, and to have, with [Intervention Author] and maybe say ‘listen, we need you to explain this to us all again’.*

(Change Agent, Ysgol-y-Cwm)

Hence, after the first reinvention point and prior to any implementation being undertaken, the intervention had started to be adapted. Although change agents indicated a future intention to expand implementation to the whole programme, a training experience that foregrounded the student support group and provided inadequate expertise in other elements ensured that the groups and their associated organisational referral mechanisms and evaluation were privileged from the outset.

### Intervention Assessment

The second reinvention point involves organisational assessment of an intervention, in order to ensure its congruence with contextual needs and resources. The characteristics determining the degree of intervention acceptability and the extent of reinvention undertaken include trialability, observability, relative advantage and compatibility (Rogers 2003). These are not objective intervention properties, however, but subjective and value-laden constructs that are determined in relation to the adoptive context, with the intervention potentially being modified to enhance acceptability and ensure contextual fit (Greenhalgh et al. 2004). In the case of the SAP, senior school managers were responsible for intervention assessment on receipt of knowledge from change agents, who sought to persuade them to adopt the programme. Change agents generally conveyed their enthusiasm for the student support group, amidst some intention to expand the intervention to encompass all 12 components at a later stage. However, through senior managers’ assessment of the SAP’s characteristics, it became apparent that this component, along with the organisational referral system and evaluation, would be the only elements to be adopted as they were most congruent with the SEL needs of the schools.

Firstly, schools’ limited resources combined with multifarious competing demands ensured that they wanted an SEL intervention that could be quickly trialled, with outcomes being easily observed. The full SAP model evaded such a rapid process of trialling, as it demanded long-term contextual restructuring. Equally, senior managers were reliant on change agents’ ability to demonstrate the feasibility and impact of the intervention. With the intervention training providing limited technical knowledge to engender structural change from the outset, these individuals were positioned to only trial the student support group. The group provided observable results, which meant that it became both the impetus and focus of further investment:

*The success of the SAP group has been the criteria used to sort of further* it.

(Senior Manager, Ysgol-y-Glyn)

…*obviously the success rate from the first group obviously helped.*

(Change Agent, Ysgol-y-Cwm)

Secondly, the SAP was relatively economically advantageous (Rogers 2003). For senior managers, there was a clear economic incentive to adopt this intervention as opposed to other SEL programmes available in the market, as local education authorities offered financial support for intervention training and resources for delivery of the student support group. This incentivisation was reflected in one senior manager’s account of their decision to adopt the programme:

*Researcher: Do you think the Local Education Authority being able to pay for the SAP training made a difference in terms of the school?*

*Senior Manager: Definitely, because our budget has definitely been reduced over the last few years. So, um, you know it’s highly beneficial because we’re also been taking part in SEAL you know as well…Um, and the LEA paid for that through sort of a funding stream. But uh, yes it does help that if it’s funded.*

(Senior Manager, Ysgol-y-Glyn)

The student support group was also deemed to offer a more efficient provision of care than existing pastoral services and could reduce the unnecessary utilisation of costly approaches such as counselling, which often received inappropriate referrals due to the dearth of provisions addressing social and emotional competencies. This was reflected in one staff member’s account of their enthusiasm for the intervention:

*I was a lone counsellor working in a school with a massive work load. And I didn’t feel I could cope with the number of young people who were being referred to me from the school because I worked in Ysgol-y-Foryd. And I could feel that the, a lot of young people were being referred to me because of behaviour, because of low attendance, and even to the point where they were using the counselling as a return to school part of a package…I selfishly though I could do more with eight than one at a time.*

(Change Agent, Ysgol-y-Foryd)

Equally, the support group was considered to reduce the burden on classroom teachers in providing social and emotional support. Indeed, teachers were invariably required to deliver more than the formal curriculum and provided an extra service through the identification and support of students’ problems. However, the extensive and often competing demands placed on staff time meant that there were limited opportunities to conduct this service. The support group could take over this provision, as a senior manager in Ysgol-y-Glyn reflected:

*It allows that staff time, because if you ware sort of teaching six lessons a day for example and you know that. There was a little boy in my class last week, come in and looked like he had the weight of the world on his shoulders. He was sort of very dirty, very unkempt, and it’s very difficult perhaps if you’ve got for fifty minutes and then he’s got to go to music or RE or whatever and the next fifty minutes.*

(Senior Manager, Ysgol-y-Glyn)

Thirdly, there was a perceived compatibility between the aims of the SAP and the discourses framing senior managers’ interpretation of the purpose of educational institutions and practices (Rogers 2003; Zins et al. 2004). While schools espoused one of a number of discourses, which focused on discipline, academic success or pastoral care (Evans et al. 2014a), these discourses tended to militate against the adoption of the full model. For illustrative purposes, the discourse of care is discussed. In Ysgol-y-Glyn, emphasis on the pastoral aspects of education was the result of the social and economic problems that were endemic, though not particular to the region. Senior managers commented on how the chaotic contexts of students’ lives meant that they were regularly deprived of SEL. Schools sought to fill this void by creating a safe space within this chaos and nurturing students’ development.

However, while schools’ orientation to the construction of a caring environment resonated with the objectives of the SAP, it posed some limitations. Essentially, the predominance of SEL meant that the SAP was forced to compete with an already overcrowded programme of intervention in the school, which included the SEAL programme, and as a result, it was reduced to its most unique and differentiated component, namely the student support group:

*SEAL is sort of seen as a whole school, um, way of talking about issues you know. Whether it’s sort of about bullying, making friends. Um, you know, whatever issues sort of come up in the general school life. We then use SAP to sort of target people…And we also then have for those pupils who’ve got sort of, who are very problematic, we’ve got counselling and we’ve got a behaviour support teacher who sees the pupils individually. So it’s whole school, groups and individuals. Depending on the need.*

(Senior Manager, Ysgol-y-Glyn)

Taken together, these sources of acceptability served to structure additional reinvention and any earlier indication by change agents that they would work to implement further elements were eventually dispensed with, in favour of focusing on the support group and its organisational referral mechanisms and evaluation.

### Intervention Clarification

The third reinvention point comprises the communication and clarification of the programme to the wider organisation. The clarification period provides an opportunity for institutional members to become accustomed to the adopted programme, understand how it fits with the broader organisational culture and become clearer about what implementation entails (Rogers 2003). Without this period of clarification, a new intervention can be interpreted as undue influence, which diminishes the incentive to change (Humphrey et al. 2010). Equally, if the staff membership is not engaged at an early stage of implementation, their commitment to delivery may be low (Zins et al. 2004).

In the case of the SAP, the entrance route of the intervention into schools via change agents and senior managers meant that the clarification period was largely absent. This was partly exacerbated by organisational constraints around communication, and change agents’ perceived lack of opportunity to discuss SEL and other educational needs outside of the formal academic curriculum. The consequence of limited clarification was that it failed to arouse an emotional investment in the SAP amongst staff. This attachment is vital, for Rogers (2003) states that a new programme must inspire a sufficient emotional reaction if it is to displace existing approaches or, at the very least, be accommodated in addition to them. However, in the absence of clarification, staff felt that schools were not committed to investing in the programme, and as a result, it was perceived to be a transient activity that would be quickly disbanded:

I *didn’t feel that it was actually wanted in the school at the time. I fancied that people were ‘Oh, something else to try out, it’s not going to work’.*

(Change Agent, Ysgol-y-Dyffryn)

*But um, to be honest not enough of the staff know enough about it, because we would say sometimes ‘We’re going to do SAP’ and some of the teachers would like roll their eyes ‘Arh, it’s just another thing which the school have put money into and it’s probably not going to work’.*

(Change Agent, Ysgol-y-Glyn)

As a result, school staffs were resolutely detached from the programme to avoid any wasted investment of time or effort. Change agents sought to circumvent such problems through the development of a second-order implementation model that exempted the broader staff membership from being involved with the student support group, which meant that efforts to introduce organisational referral mechanisms and staff evaluation were quickly disbanded. Indeed, staffs were merely requested not to disrupt or obstruct delivery. This approach led to a loss of traction, as the intervention was further reinvented as a peripheral student support group that was located at the fringes of educational practices and priorities, and was largely elided within the everyday activities of the school.

### Intervention Responsibility

The final reinvention point influences decision-making around the confirmation or discontinuance of a programme and the extent to which it becomes a routinised part of organisational practices. It captures the process of assigning responsibility and accountability, either explicitly or implicitly, for long-term intervention sustainability. In the case of the SAP, previous reinvention points ensured the burden of intervention delivery lay with a limited number of change agents, with the majority of school staff being exempt from responsibility. Over-reliance on these individuals during diffusion and delivery led to intervention burnout. This problem was exacerbated by emotional detachment amongst staff, combined with limited organisational capacity. For one change agent, frustration at a lack of support for the student support group had left them disillusioned and reluctant to continue to drive implementation:

*Andrea said she felt exhausted and couldn’t wait for the end of term. The groups hadn’t been successful and it had worn her out. She had experienced some issues with teachers who were supposed to deliver the group. The problem was that they currently had to give up their preparation lessons in order to run the group, but this left them without any time to prepare for their normal lessons… She said the school was never going to be a Centre of Excellence if they were not prepared to release teachers, and she was fed up with trying. She was no longer sure what the future of SAP would be.*

(Field notes, Ysgol-y-Dyffryn)

As a consequence of such experiences, these individuals often renounced responsibility ensuring that during this reinvention point, the intervention was discontinued in three schools.

## Discussion

Implementation has proved to be a major challenge to public health intervention, and flawed diffusion strategies have compromised the effectiveness of a number of SEL programmes (Durlak and DuPre 2008; Durlak et al. 2011; Humphrey et al. 2010). This paper introduces the concept of reinvention points, which may serve as a useful theoretical device in understanding the complexity of intervention delivery within real-world settings, while illustrating how marked variations in implementation practices are often the consequence of incremental, subtle and even unconscious decisions to adapt an intervention. Reinvention may be defined as the refinement or transformation of an intervention through its interaction with individual agents and contextual features. This paper identified four key reinvention points that contributed to the SAP being reduced to a peripheral student support group with no organisational referral mechanisms or staff support, before being discontinued. These reinvention points include the following: intervention training, which captures the process through which adopters acquire awareness of a programme; intervention assessment, which reflects adopters’ evaluation of an intervention in relation to contextual needs; intervention clarification, which comprises the cascading of knowledge through an organisation to secure support in delivery; and intervention responsibility, which relates to the extent to which individuals are empowered to ensure sustainable delivery.

Diffusion of innovations theory (Greenhalgh et al. 2004; Rogers 2003), which has come to underpin a number of implementation frameworks (Spoth et al. 2013; Wandersman et al. 2008), serves as the overarching theoretical frame for conceptualising reinvention points. As a dynamic, causative model, this theory charts the different needs that individuals and organisation will express and experience at different times during intervention delivery. The reinvention points identified in this paper can be mapped onto Rogers’s (2003) stages of diffusion and are expressed during the transition between these phases as this is where intervention practices are most in flux and where programmes may be most susceptible to individual needs and contextual influences, which are themselves changeable as they response to intervention reinvention (Elliott and Mihalic 2004).

The foregrounding of the diffusion of innovations, and reinvention points as part of this, encourages reflection on the functionality of existing methodological and evaluative frameworks in capturing the complex determinants and consequences of implementation. Process evaluations present the primary evaluative mechanism for measuring implementation. Although there remains no overarching approach, guidance has recently been published by the Medical Research Council (Moore et al. 2014). Yet, even with a historical lack of consensus, Linnan and Steckler’s (2002) criteria and Glasgow et al. (1999) RE-AIM (reach, effectiveness, adoption, implementation and maintenance) framework have gained prominence. However, these remain limited by their measurement of discrete intervention activities (e.g. reach or adherence) rather than processes, failing to illuminate the mutually determined relationships between constituent parts, as identified by diffusion of innovations theory and the concept of reinvention. This does not preclude the nesting of implementation checks within these frameworks in order to assess levels and variation in fidelity. Indeed, a number of comprehensive and conceptually coherent assessment tools are in operation, and these are vital in linking delivery to outcomes in order to circumvent type 3 errors (Carroll et al. 2007). Rather, these checks need to be located within a wider context of understanding.

Reinvention points also contribute to the theoretical and empirical insights offered by implementation science, while highlighting additional opportunities to predict and intervene with implementation problems within real-world settings. Firstly, the initial reinvention point of training reveals an evident need for high quality training that offers extensive knowledge and technical expertise, combined with continued support for diffusion activities (Fixsen et al. 2005; Greenberg 2010; Wandersman et al. 2008). Indeed, all four reinvention points demonstrate the necessity of both intervention developers and funders providing substantial and continued training beyond a brief 3-day course. Yet, despite the apparent inadequacies of the SAP training, the impact of simulation activities did encourage extensive commitment and passion amongst change agents, and as enthusiasm for a programme’s concepts and aims is linked to fidelity (Dane and Schneider 1998), the integration of experiential learning into training may be appropriate. However, this learning should not be offered at the cost of acquiring technical expertise.

Secondly, the reinvention point of persuasion highlights how presumptions around cultural specificities or institutional idiosyncrasies may lead to adaption as organisations assess interventions in terms of trialability and observability, relative advantage, and compatibility (Rogers 2003). Within the SAP, this process was conducted by senior managers who were supported by knowledge from change agents. The implications of this reinvention are that in addition to substantial training for change agents, it is imperative that sufficient information and expertise are provided to organisational leaders so that they know how to best support implementation. Perhaps more importantly, it is necessary to engage those in leadership positions in debates around functionality, emphasising the fact that while some variation in composition may be permitted to accommodate contextual needs, theoretical integrity must be preserved (Hawe et al. 2004). To this end, the sharing of intervention logic models may be of use, so there may be increased understanding of where reinvention may negate impact.

Thirdly, reinvention at the point of clarification suggests that over-reliance on an individual intervention champion, or a small number of champions, may not provide the most appropriate diffusion mechanism, despite regular recommendations for their presence within the literature (Elliott and Mihalic 2004). The change agents within the SAP routinely encountered a number of structural barriers to communication, which prevented them from sharing the requisite knowledge and securing colleagues’ involvement, ensuring the SAP’s increasing peripheral position within schools. There is clearly a need at this point to ensure organisational capacity for the clarification period where there are sufficient resources and administrative support (Humphrey et al. 2010; Rogers 2003). Additionally, as illustrated elsewhere, there is a requirement for strong and supportive leadership, particularly with regard to creating a positive climate where SEL is privileged and prioritised amongst staff (Durlak and DuPre 2008; Kam et al. 2003). Equally, it is important to ensure adequate training to all staff members expected to be involved in delivering any aspect of the intervention, rather than relying on the cascading of knowledge by change agents, as there is clear evidence to suggest that adherence, or ‘treatment integrity’, is higher following direct rather than indirect training methods (Sterling-Turner et al. 2002; Durlak and DuPre 2008).

Fourthly, the final reinvention point of intervention responsibility, which highlights the phenomenon of intervention burnout amongst key change agents, suggests the need to refocus existing prescriptions for sustainable intervention practice. Although there have been calls to provide support and resources to individual implementers within schools (Kam et al. 2003; Zins et al. 2004), there may be an additional need to distribute the implementation burden more evenly across the system so that burnout, and hence discontinuance, may be avoided (Bond et al. 2004).

### Limitations

The study is limited by the small number of cases that contributed to the generation of data. Although the four schools were not untypical of secondary schools within Wales, local educational authorities were selected for being the most socio-economically diverse regions implementing the intervention. However, despite limitations to generalisability, the case study data are instructive in the development of theoretical propositions that may structure the exploration of implementation procedures in other educational contexts (Yin 1994).

## Conclusion

Implementation remains a substantial challenge for public health interventions. While there has been a proliferation of frameworks that seek to quantify adherence, there remains a limited range of models to understand the complex determinants of implementation practices. Diffusion of innovations theory offers a comprehensive and nuanced theoretical lens, as it shifts the locus of interest to the interaction of interventions with contextual features and individual needs, and how this transformative process gives rise to programme reinvention. Four key reinvention points were conceptualised in this study: intervention training, intervention assessment, intervention clarification and intervention assessment. Taken together, these reinvention points illustrate the challenge of implementing complex intervention, demonstrating the need for developers and funders to invest in substantial and continued training, in addition to ongoing technical assistance. They equally suggest opportunities to predict and intervene with potential implementation problems while serving as a useful departure point for exploring additional reinvention activity across the phases of diffusion.
